# The Engineering Side of Hospital Work

**Published:** 1905-11-18

**Authors:** 


### THE ENGINEERING SIDE OF HOSPITAL WORK.

### XV.—ELECTRICAL APPARATUS.

(*Continued from page* 92.)

The Galvanic Battery.

As already explained, a galvanic battery is formed whenever two dissimilar substances are present, and particularly when two dissimilar metals are present with a liquid, and there is a path for any current that is generated. For practical use, however, there are only two forms of galvanic battery, the Le Clanchè and the bichromate. The former is used for bells and telephones, and has been used for providing continuous currents for medical purposes and for small induction coils; the latter is used principally where a powerful current is required for surgical purposes. In all practical galvanic batteries, there are one or more zinc and one or more carbon plates. The zinc stands in a solution of sal ammoniac in the Le Clanchè, and in a solution of sulphuric acid and bichromate of potash, or chromic acid, in the bichromate. The zinc is always eaten away as the battery works, and requires renewing as it wears. In the bichromate cell also it requires cleaning, and sometimes reamalgamating after use, the action of the salts in the solution in which it is immersed forming a deposit upon the surface of the zinc which not only opposes the passage of the current, but sets up an opposing electrical pressure to the primary current. In both the Le Clanchè and the bichromate cells hydrogen is given off at the carbon plate, and would again lessen the current by setting up resistance and opposing pressures unless it was got rid of. In the Le Clanchè the hydrogen is got rid of by providing some oxide of manganese for it to attack, the latter giving up a portion of its oxygen rather freely, and the hydrogen uniting with the oxygen to form water. The oxide of manganese is contained in a porous cell, in which also the carbon plate stands, and in which some crushed carbon is placed with the manganese. In the dry cells, which are all on the Le Clanchè pattern, the solution of sal-ammoniac is held in plaster of Paris, or some similar substance, the whole being sealed over. There are very few dry cells that are of any use, and none of small size. In the bichromate cell, the oxidising agent is bichromate of potash, or chromic acid, both of which give up their oxygen rather freely. The bichromate cell is sometimes made with a porous division, the carbon plates standing in the outer portion of the cell, the zinc in the porous cell, but the presence of the porous division so increases the resistance of the cell that it is rendered almost useless for cauteries, or work requiring large currents. The more usual form where large currents are required is, the zinc is fixed between two carbons, and the three are arranged to be lifted out of the solution when not in use, and to be immersed sufficiently to give the necessary current. The pressure available from any battery cell depends, first upon the constituents of the battery, and secondly upon the internal resistance. The chemical constituents and their arrangement rule the highest pressure obtainable, which is obtained when no current is passing. The resistance of each cell takes toll of the initial pressure, in proportion to the current passing, the actual toll being measured by the product of the resistance into the current. The resistance of each cell depends upon its dimensions. The larger the plates in the cell, and the nearer they are togther, the smaller the resistance, hence the "plunge" bichromate has a low resistance, compared to the Le Clanchè, and hence also its available pressure is higher than that of the Le Clanchè, and it will be remembered that the heating effect depends upon the square of the available pressure.

Primary batteries are convenient, but if required to furnish much current, and for long, give a great deal of trouble, especially where they are small. The staying power of a galvanic cell depends upon the quantity of solution in which the zinc is immersed, and upon the quantity of the oxidising agent present. The presence of a large quantity of the zinc solution, tends to keep the surface of the zinc clean, by dissolving off the salts that are formed, and the presence of a large quantity of the oxidising agent necessarily adds to the staying power, because it neutralises the hydrogen gas more quickly and for longer. Small cells should never be used, except in very special cases. They will always give trouble, because they cannot contain large quantities of either of the substances named. Portable cells are usually a snare. Small cells may in very careful hands give good results, but it will only be where they receive very great attention. Small Le Clanchè or dry cells never can answer, except for a very short time after they are first made up. For telephone- and bell- and similar work some few dry cells answer well. The writer knows of two which give as good results as any open Le Clanchè of large size, one a German and the other a French make. The text-books, even to this day, contain instructions showing how larger currents may be obtained from small cells by connecting them in what electrical engineers call parallel circuit. Instead of connecting the cells tandem—the zinc of one cell to the carbon of the next, and so on, leaving a zinc unconnected at one end and a carbon at the other—they are to be connected abreast, all the zincs being connected together and all the carbons, or in the series-parallel method, the end carbons of a series with the end carbons of all the other groups. The method is quite wrong. It will not always give additional current and it always causes the cells to exhaust very much quicker than they would do if connected in series. The reasoning upon which the parallel method is based is, the pressures created by each cell and by each series of cells is supposed to be exactly the same, and so each cell or group is supposed to bring its quota of current. In practice no two cells and no two groups of cells have the same pressure or resistance. Hence, when they are joined in parallel, the cell or the group which has the highest pressure alone delivers current, while the other cells form additional paths for the current from the cell whose pressure is highest. This leads to the cells which commence delivering current being worked down very quickly, and then another cell or group takes up the running with the same result, and so on. The outer circuit is only the better for a short time, if at all, and the whole of the battery runs down very quickly. All batteries should be kept very dry for a similar reason. The moisture on the outside forms a path for the current, which is always open, and is working the cells down. If primary batteries must be used for large currents or hard, continuous work, make the cells as large as possible. Doubling the size of a cell usually trebles its staying power.

